# Extracellular Microenvironmental Change by B16F10 Melanoma-derived Proteins Induces Cancer Stem-like Cell Properties from NIH3T3 Cells

**DOI:** 10.1038/s41598-019-53326-8

**Published:** 2019-11-14

**Authors:** Soon Yong Park, Dong Gwang Lee, Ara Jo, Hyeongrok Choi, Joo Eon Lee, Ae Jin Jeong, Sun-Hee Leem, Woojin Jun, Sangin Shim, Sang-Kyu Ye, Jeong-Ki Min, Jin Woong Chung

**Affiliations:** 1 Department of Biological Science, Dong-A University, Busan, 49315 Republic of Korea; 2 Biotherapeutics Translational Research Center, Korea Research Institute of Bioscience and Biotechnology, Daejeon, 34141 Republic of Korea; 3 Department of Biomolecular Science, University of Science & Technology, Daejeon, 34141 Republic of Korea; 4 Division of Drug Development and Optimization, KBIO-New Drug Development Center, Cheongju, 28160 Republic of Korea; 5 Department of Pharmacology and Biomedical Sciences, College of Medicine, Seoul National University, Seoul, 03080 Republic of Korea; 6 Department of Food and Nutrition, Chonnam National University, Gwangju, 61186 Republic of Korea; 7 Department of Agronomy, Gyeongsang National University, Jinju, 52828 Republic of Korea

**Keywords:** Cancer microenvironment, Cancer stem cells

## Abstract

Cancer stem-like cells (CSCs) can generate solid tumors through the properties of stem cells such as self-renewal and differentiation and they cause drug resistance, metastasis and recurrence. Therefore, establishing CSC lines is necessary to conduct various studies such as on the identification of CSC origin and specific targeted therapies. In this study, we stimulated NIH3T3 fibroblasts to exhibit the characteristics of CSCs using the whole protein lysates of B16F10 melanoma cells. As a result, we induced colony formation that displayed self-renewal and differentiation capacities through anchorage-independent culture and re-attached culture. Moreover, colonies showed drug resistance by being maintained in the G0/G1 state. Colonies expressed various CSC markers and displayed high-level drug efflux capacity. Additionally, colonies clearly demonstrated tumorigenic ability by forming a solid tumor *in vivo*. These results show that proteins of cancer cells could transform normal cells into CSCs by increasing expression of CSC markers. This study argues the tremendous importance of the extracellular microenvironmental effect on the generation of CSCs. It also provides a simple experimental method for deriving CSCs that could be based on the development of targeted therapy techniques.

## Introduction

Cancer stem-like cells (CSCs) are cancer cells that have the abilities of normal stem cells, such as self-renewal and differentiation into various types of cancer cells. Therefore, CSCs can form a solid tumor through self-renewal and continuous differentiation^1^. CSCs with these abilities are distinguished from other cells in the tumor, and are the major cause of metastasis, drug resistance and recurrence in many solid tumors^2^.

CSCs were found in various solid tumors, such as bladder, breast, brain, colon, prostate, pancreatic cancer, and melanoma after the first CSCs had been found in acute myeloid leukemia in 1997^3^. CSCs are present in small amounts in cancer tissues, but they possess great properties that can cause drug resistance, metastasis, and recurrence^2,4–6^, leading to higher lethality of cancer patients even with advanced chemotherapy and radiotherapy. Consequently, CSC-targeted therapies are necessary for a more effective anti-cancer therapy. However, CSC research is not well studied because CSCs are difficult to obtain, the culture method has not been clearly established, and they should be isolated directly from the primary tumor tissue.

Although the hypothesis that tumors originate from stem cells was already proposed in 1875^7^, the mechanism of the generation of CSCs has still not yet been clearly demonstrated. However, three hypotheses have been suggested on the CSC generation models up to date^1,8^. First, most tumors consist of a low multipotent daughter cells produced from CSCs. Mutations accompany genome damage during asymmetric cell division in stem cells and it develop into a transformed state as CSCs. Then, not like normal stem cells, these CSCs develop into a tumor that contains a variety of mutated cells. Second, CSCs are induced by the accumulation of gene mutations in normal stem cells. This hypothesis can be the basis for demonstrating the differentiation potential of CSCs. Third, already differentiated cells undergo dedifferentiation and acquire similar characteristics to stem cells. These stem cell-like cells undergo epithelial–mesenchymal transition, which is a key process in tumorigenesis, thus leading to CSC formation through the transformation of the stem cells^9,10^.

The third model is similar to the formation mechanism of dedifferentiated embryonic stem cells. In 2006, Yamanaka produced stem-like cells from mouse and human somatic cells called induced-pluripotent stem cells^11,12^. Yamanaka introduced four factors, namely, *Oct4*, *Sox2*, *c-Myc*, and *Klf4*, into the somatic cells using a retrovirus to form a colony morphology similar to that of embryonic stem cells, confirming that these induced colonies are similar in characteristics to embryonic stem cells.

The role of the tumor microenvironment in the CSC generation process has recently been reported. The microenvironment of normal stem cells is composed of neovasculature, extracellular matrix, and paracrine factors. Stem cells regulate self-renewal and differentiation through a specific microenvironment; similarly, CSCs also maintain their properties by interacting with the microenvironment^13–17^. Although many studies on the tumor microenvironments of CSCs are insufficient, the diverse factors in cancer cells may be major components of the tumor microenvironment. On one hand, the tumor microenvironment plays a role in regulating the growth and differentiation of cancer cells. On the other hand, oncogenes such as *c-Myc* and *Klf4* are major factors in the dedifferentiation process from somatic cells.

Thus, this study aimed to determine whether the extracellular microenvironment change by various intracellular components of cancer cells could convert mouse fibroblasts into putative CSCs. Surprisingly, we found that the treatment with protein lysates of B16F10 melanoma cells could transform NIH3T3 cells into the colony form, which possessed the characteristics of CSCs.

## Results

### B16F10 melanoma cell-derived proteins induce colony formation in NIH3T3 cells

**Figure 1:**
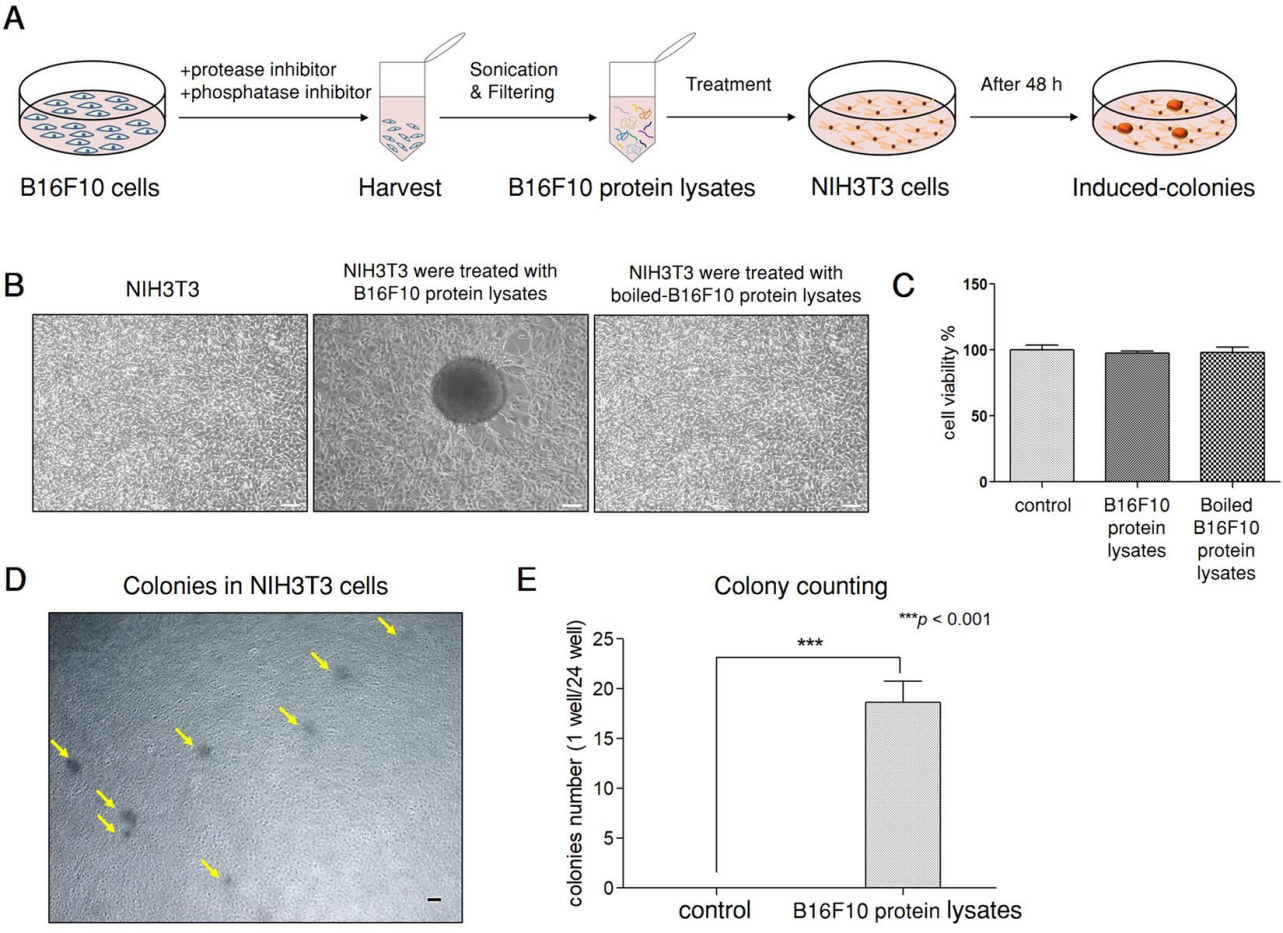
B16F10 melanoma-derived proteins can induce colony formation from NIH3T3 cells. (**A**) Colony formation induction model using the proteins of cancer cells from normal cells. (**B**) Microscopic analysis of the induced colony formation from NIH3T3 cells. The NIH3T3 cells were treated with the B16F10-derived proteins and heat-inactivated proteins (50 μg/ml) for 48 h. (**C**) Measurement of cell viability after the treatment of cancer cell-derived and heat-inactivated proteins for 48 h (n.s: no significant). Cancer cell-derived proteins did not affect the viability of the NIH3T3 cells at the designed concentration. (**D**,**E**) About 15–20 colonies were induced in one well of the 24-well plates from the NIH3T3 cells by the B16F10 proteins 50 μg/ml (yellow arrow). These results are the averages of three independent experiments (***P < 0.001). Scale bar: 100 μm.

To investigate the colony-inducing effect of cancer cell-derived proteins on mouse fibroblast NIH3T3 cells, we first treated the B16F10 cell-derived proteins on the NIH3T3 cells, and observed morphological changes in the fibroblast. Interestingly, the NIH3T3 cells treated with B16F10 cell-derived proteins induced a colony formation in only 48 h (Fig. 1A). We observed a definite induction of colony formation by the B16F10 cell-derived proteins, while the boiled B16F10 cell-derived proteins could not induce colony formation in the NIH3T3 cells (Fig. 1B), suggesting that the major factors for the colony formation are the proteins in the cell lysates. Then, we next produced NIH3T3-GFP stable cells to prove that the colonies were originated from the NIH3T3 cells (Supplementary Fig. S1A,B). Furthermore, the 50 μg/ml of B16F10 cell-derived proteins did not affect the cell viability on the treated NIH3T3 cells (Fig. 1C). However, the cell viability was decreased in a concentration-dependent manner from 100 μg/ml or more (Supplementary Fig. S1C). Moreover, 12–20 colonies were generated in one well of a 24-well plate (Fig. 1D,E, Supplementary Fig. S1D,E). These results suggest that the B16F10 cell-derived proteins with a proper concentration quickly induce colony formation, which is a specific characteristic of stem cells, and do not affect survival in normal mouse fibroblast NIH3T3 cells.

### Induced colonies by the B16F10 melanoma cell-derived protein treatment acquire the properties of stem cells

**Figure 2:**
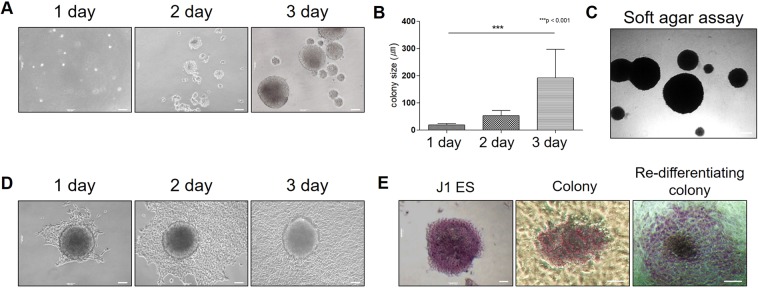
Induced colonies acquire the properties of stem cells. (**A**,**B**) The induced colonies formed a spheroid morphology and grew on ultra-low attachment plates. (**C**) The induced colonies also formed spheroids and maintained their morphology in a soft agar medium. (**D**) The anchorage independently cultured induced colonies were transferred to normal culture plates, and the colonies were differentiated into normal cells in a time-dependent manner. (**E**) The induced colonies were stained with AP staining solution, but the stained areas faded and spread out of the colonies as the differentiation progressed. These results are the averages of three independent experiments (***P < 0.001). Scale bar: 100 μm.

To determine whether the induced colony possessed the characteristics of stem cells, we observed the spheroid formation ability in a single cell of the colony on ultra-low attachment plates in a mES medium. The single cell of the colony formed spheroids effectively, and the growth of spheroids accelerated in a time-dependent manner (Fig. 2A,B). The generated colonies from the NIH3T3-GFP stable cells could also form GFP-positive spheroids (Supplementary Fig. S2A). Furthermore, we conducted a soft agar colony formation assay to evaluate the anchorage-independent growth ability of induced colonies. We also observed that induced colonies formed remarkable spheroids in soft agar. Thus, the induced colonies acquired the self-renewal ability of stem cells (Fig. 2C). Then, we investigated the differentiation ability of colonies *in vitro* by re-attaching them to the culture plates. The re-attached colony on the culture plates re-differentiated to normal cells and grew over time (Fig. 2D). In addition, the re-attached GFP-positive colony could re-differentiate and proliferate (Supplementary Fig. S2B). We then performed the AP staining test to identify the alkaline phosphatase activity, which is a characteristic of stem cells. The colony was positively stained in circular form, and the stained area was blurred to the outside of the edges from the colony over time (Fig. 2E). Taken together, these experiments provide substantial experimental evidence to support the concept that proteins from cancer cells could construct a tumor microenvironment that induces dedifferentiation and re-differentiation capacities in normal cells.

### Induced colonies acquire the properties of CSCs

**Figure 3:**
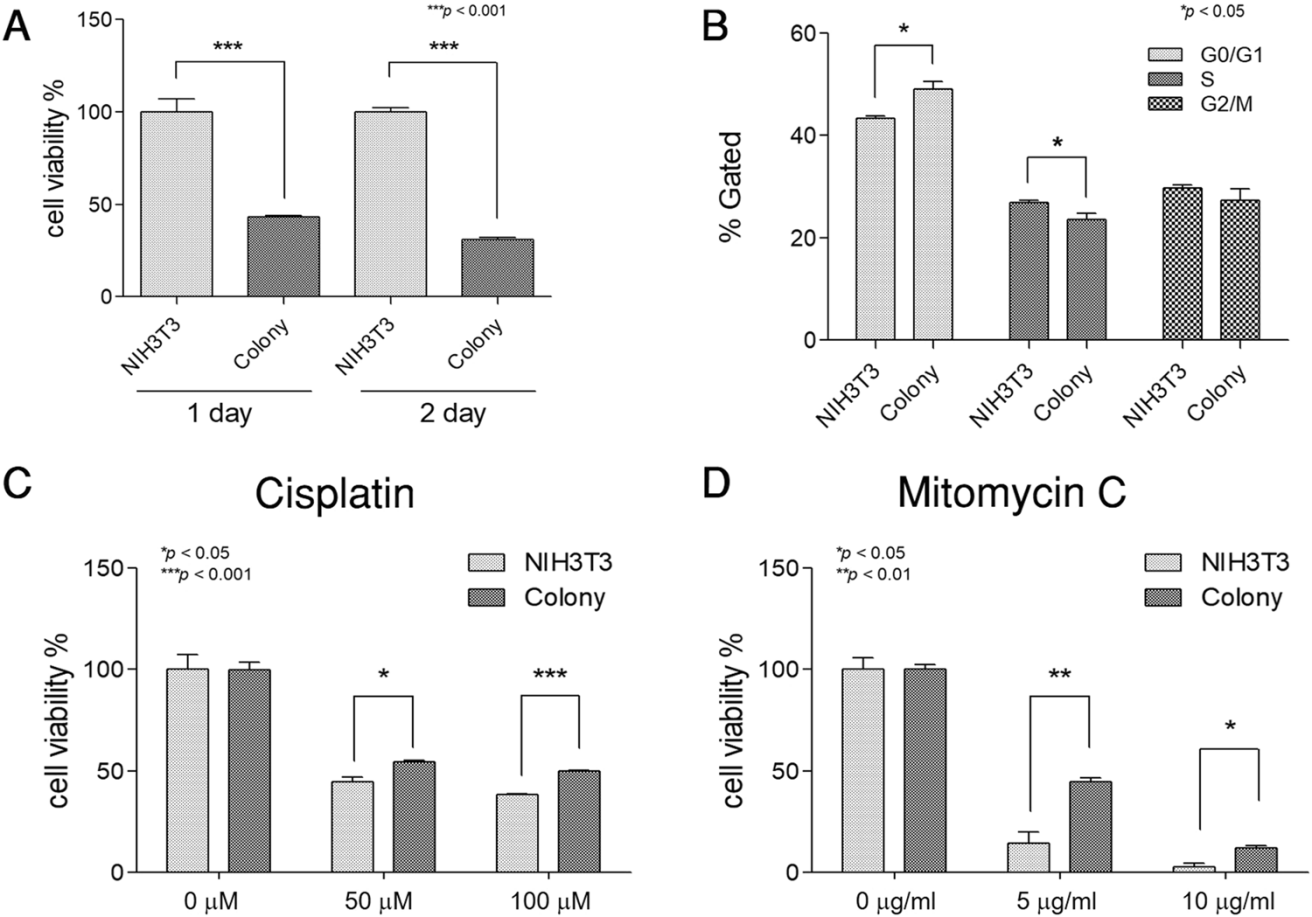
Induced colonies acquire the specific characteristics of CSCs. (**A**) Cell proliferation rate measurement of the induced colonies and the NIH3T3 cells by MTT assay. Growth speed of the induced colonies was slower than that of the NIH3T3 cells. (**B**) Cell cycle distribution analysis of the induced colonies and the NIH3T3 cells using the flow cytometry. The colonies had a higher proportion of the G0/G1 phase and a lower proportion of the S phase and G2/M phase than the NIH3T3 cells. (**C**,**D**) The induced colonies and the NIH3T3 cells were treated with cisplatin and mitomycin C at a designed concentration. Cell viability of the induced colonies was higher than that of the NIH3T3 cells. These results are the averages of three independent experiments (*P < 0.05, **P < 0.01, ***P < 0.001).

In general, CSCs have a slower proliferation rate than normal cancer cells to maintain a high survival level of the tumor against diverse chemotherapies. To determine whether the induced colony has the capabilities of CSCs, we measured the proliferation levels of the NIH3T3 cells and the induced colony. The growth rate of the induced colonies was two times lower than that of the NIH3T3 cells (Fig. 3A). Then, we analyzed the distribution of the cell cycle phase in the NIH3T3 cells and the induced colonies because CSCs retain a high ratio of the G0/G1 phase. The percentage of the G0/G1 phase cells was higher in the induced colonies than in the NIH3T3 cells (Fig. 3B, Supplementary Fig. S3). Thus, as expected, the induced colonies maintained a high level of G0/G1 phase similar to the CSCs. In light of the recent studies showing that cells with a high level of G0/G1 could resist anti-cancer drugs, we measured the survival rate of the NIH3T3 cells and the induced colonies against anti-cancer drugs, such as cisplatin and mitomycin C, through the MTT assay. Resistance against anti-cancer drugs was confirmed by the cell viability at the same concentration and time point, showing that the colonies were more resistant to cisplatin and mitomycin C than the parental NIH3T3 cells (Fig. 3C,D). Our data demonstrated that the colonies induced by the B16F10 cell-derived protein treatment in the NIH3T3 cells obtained resistance against cisplatin and mitomycin C. This observation indicates that induced colonies acquire the properties of CSCs such as anti-cancer drug resistance.

### Induced colonies express the major markers of CSCs

**Figure 4:**
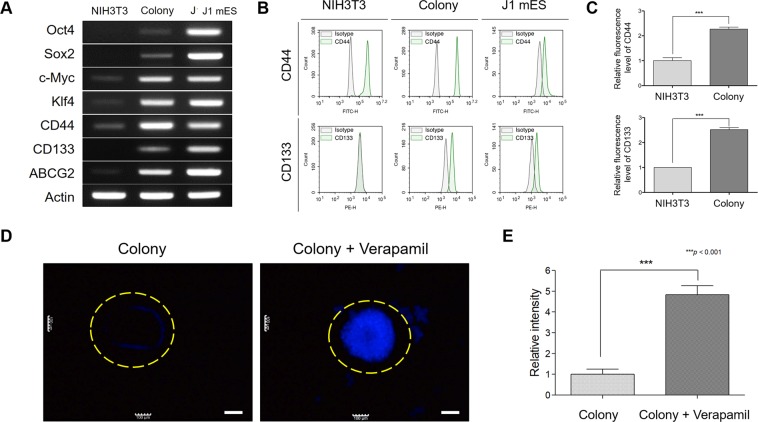
Various markers of CSCs are expressed in induced colonies. (**A**) RT-PCR analysis was performed to examine the expressions of specific markers. The stem cell markers *Oct4*, *Sox2*, *c-Myc*, and *Klf4* and the CSC markers *CD44*, *CD133*, and *ABCG2* were activated in the induced colonies (full-length gels are presented in Supplementary Fig. S4). (**B**,**C**) Expression levels of CD44 and CD133 proteins in the induced colonies and the NIH3T3 cells. The CD44 and CD133 protein expressions were significantly elevated in the induced colonies in comparison with the NIH3T3 cells. (**D**,**E**) Activation of the efflux function was measured to investigate the efflux level, that is, the chemotherapy resistance capacity. The non-treated colonies were hardly stained, whereas the verapamil-treated colonies were completely stained by Hoechst 33342. These results are the averages of three independent experiments (***P < 0.001). Scale bar: 100 μm.

We examined whether the induced colonies could express the key markers of CSCs and stem cells. We performed RT-PCR to confirm the marker expressions not only of stem cells, such as Oct4, Sox2, c-Myc, and Klf4 but also of CSCs, such as CD44, CD133, and ABCG2. We observed an increase in the expression of Oct4, Sox2, c-Myc, Klf4, and ABCG2 in the colonies. Moreover, the CD44 expression significantly increased in the colonies compared with the NIH3T3 cells, and the CD133 expression was induced by the B16F10 cell-derived proteins. Interestingly, the NIH3T3 cells did not express Oct4, Sox2, and CD133, but the induced colonies expressed them (Fig. 4A). To determine whether the expressions of CD44 and CD133 increased in the colonies at the cellular level, we performed a flow cytometry analysis to measure the expression of the cell surface markers. As expected, the expression levels of CD44 and CD133 were significantly amplified in the colonies compared with the NIH3T3 cells (Fig. 4B,C). To evaluate whether the increased expression of ABCG2 could resist the cytotoxicity of anti-cancer drugs, we observed the level of efflux capacity of the colonies using verapamil as the ABC transporter inhibitor. The colonies pre-treated with verapamil were completely stained with Hoechst 33342 dye, but the untreated colonies were weakly stained at the edges (Fig. 4D,E). This observation indicates that the induced colonies acquire the CSC attributes through the induction of specific markers, such as Oct4, Sox2, ABCG2, and CD133. Consequently, the increased expression of these factors enables the expansion of the self-renewal ability and drug resistance capacity by regulating the cell cycle.

### Induced colonies form tumors in nude mouse

**Figure 5:**
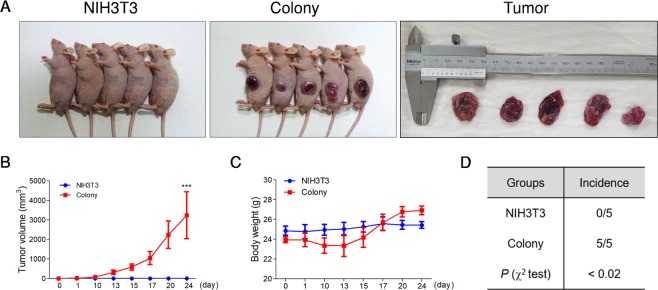
Induced colonies can generate a solid tumor *in vivo*. (**A**–**D**) Xenograft assay was performed to verify the tumorigenic capacity of the induced colonies. Each of the five BALB/c nude mice was injected subcutaneously (s.c.) with 1 × 10^6^ NIH3T3 cells and the induced colonies. All five mice injected with the colonies formed and developed solid tumors, whereas all the five mice injected with the NIH3T3 cells did not form solid tumors. These results are the averages of three independent experiments (***P < 0.001).

To verify further the tumor formation potency of the induced colonies *in vivo*, the NIH3T3 cells and the colonies were injected into the subcutaneous of BALB/c nude mice. For 24 days, five out of the five mice (100%) injected with colonies developed and grew measurable tumors, whereas zero of the five mice (0%) injected with the NIH3T3 cells formed any tumors (Fig. 5A–D). Moreover, the mice injected with the colonies increased in body weight because of the growth of the tumor volume, but we did not observe any effect on the mice body weight with the injected NIH3T3 cells. This result shows that the induced colonies have similar characteristics to the CSCs and possess the ability to form solid tumors. Additionally, we compared the expression of specific factors by RT-PCR analysis to characterize the cancer cells derived from solid tumors. Surprisingly, as the passage of the tumor-derived cells (TDCs) increased, the expressions of stem cell markers such as Oct4, CD133, and Sox2 decreased rapidly while the expression levels of oncogenes including c-Myc, Klf4 and ABCG2 were maintained (Supplementary Fig. S5). This observation indicates that the induced colonies from the NIH3T3 cells maintains the tumorigenic properties such as solid tumor initiation, growth, and progression *in vivo*, retaining their differentiating capacities.

## Discussion

Owing to the robust researches focused on CSCs since its first identification in 1990s, many proofs showing important roles of CSCs in tumor progression has been accumulated, and it is now generally accepted that the CSCs may be the major cause of metastasis and recurrence of various types of cancers. However, the origin of CSCs is still controversial although several models have been proposed^18–21^.

According the cell theory, all cells arise from preexisting cells. Then, the CSCs also should arise from preexisting cells in the body. Apparently, not like normal adult stem cells, CSCs are found only in the cancer patients. This fact may strongly suggest that cancer should first arise, and somehow affect the formation of CSCs.

When tumors are initiated, a variety of physiologic conditions surrounding them are changed, thus establishing a new tumor microenvironment. In fact, the tumor microenvironment and host factors have been known to play a major role in the establishment, progression, and metastasis of cancers^22–25^. The tumor microenvironment is composed of various cells and components, such as cytokines and growth, angiogenic, and inflammatory factors released from many types of cells. Interestingly, tumor-host interaction has been reported to control the tumor microenvironment, and maintain optimal conditions for tumor survival^22–25^. In fact, tumors could maintain their specific characteristics through continuous interaction with the unique surrounding microenvironment^26,27^. Therefore, we speculated that the tumor itself may a major factor for CSC formation, and investigated the effects of tumor-derived factors on normal fibroblast, which might be a representative of cellular tumor microenvironmental factors^28^.

Unfortunately, our first attempt showed no significant changes in fibroblasts when they were co-cultured with tumor cells (Supplementary Fig. S7), suggesting that direct interaction between tumors and host cells may not be the critical factor for CSC formation. Surprisingly, however, treatment of the fibroblast with total protein lysates from the tumor cells was able to induce distinct colonies *in vitro* while heat-inactivated proteins did not affect any changes in fibroblast, indicating the specific effect to tumor-derived proteins (TDP) on colony formation. Colony formation is a specific morphological hallmark of stem cells. Therefore, we hypothesized that induced colony cells would acquire the properties of stem cells.

As expected, these colonies were positive from alkaline phosphatase staining which indicates that they have characteristics of undifferentiated stem cells. Moreover, the fact that the stained area was blurred to the outside of the edges from the colony over time shows that these colonies still possess the differentiating abilities as proved in the previous reports^11,12^.

By observing the properties of survival and proliferation in the anchorage-independent culture by using ultra low-attachment plates and conducting soft agar assay, we found that the TDP-induced colonies could survive without anchorage, thus suggesting their unique ability of self-renewal, not only as stem cells, but also as cancer cells^29–31^.

Recently, various CSC specific markers, such as ABCG2, CD44, and CD133, have been identified. In fact, the TDP-induced colonies are shown to express these CSC markers along with Oct4, Sox2, c-Myc, and Klf4, which are the major de-differentiation factors. The fact that the higher level of these markers may suggest that the TDP could induce the de-differentiation of fibroblast into stem cell like colonies, and these colonies acquired the cancerous traits, ultimately forming CSCs, which supports the previous report^32–34^.

ABCG2 is one of the ATP-binding cassette (ABC) transporter types, and activates the efflux function contributes to a strong resistance of CSCs against chemotherapy by expelling the drugs or intracellular compounds^35,36^. Actually, our results show that the colonies possess the drug efflux activity, and exhibit resistance to various anti-cancer drugs, proving their characteristics as CSCs. We also investigated the proliferation rate of the colonies in comparison with parent fibroblast cells. As expected, the proliferation of the colonies was much slower than that of the fibroblast cells. In general, normal cancer cells have a high S phase ratio and proliferation rate, whereas CSCs have a high G0/G1 ratio and can remain in the quiescent state of the cell cycle. Thus, our data showing the higher G0/G1 and lower S phase and G2/M ratio of the colonies than those of the parent fibroblast strongly support that TDP-induced colonies would acquire the characteristics of CSCs.

Ultimately, the tumor initiation ability of the TDP-induced colonies was confirmed *in vivo* through a xenograft assay using BALB/c nude mice, where the isolated cells from the colonies were able to form solid tumors. A remarkable fact is that these tumor initiating cells were originally derived from normal fibroblast cell lines just by treating them with TDPs. According to our results, the optimal concentration of proteins to accomplish the best efficiency of colony induction should be 50–100 μg/ml. Actually, incubation of the fibroblast with the culture supernatant of the tumor cells did not affect the colony formation of the parent cell lines (Supplementary Fig. S8), suggesting that the amount of the secreted proteins in the supernatant may not be enough to induce CSC formation. In fact, TDP could not induce colony formation when the concentration was less than 50 μg/ml *in vitro* (data not shown). As a matter of fact, this concentration seems to be higher than physiological conditions, that would never happen in the body. However, previous reports showed that dying cancer cells could induce the repopulation of tumor cells through caspase 3-mediated mechanisms^37^. Therefore, certain factors might be massively released during cancer therapies, including chemotherapy or radiotherapy, and possibly induce the transformation of neighboring cells into CSCs *in vivo*, although the mechanisms should be carefully studied in the future.

**Figure 6:**
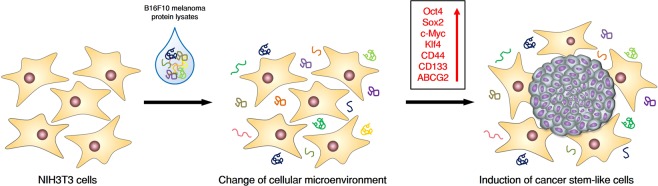
Summary. In this study, the cancer cell-derived proteins could induce colony formation from normal cells by regulating the extracellular condition. These colonies acquired the characteristics of the CSCs by upregulating the various factors related to CSCs. These colonies also obtained tumorigenic potentiality *in vivo*.

In summary, we first found that tumor-derived protein is one of the major factors in CSCs generation, possibly by inducing dedifferentiation of neighboring cells surrounding the tumors (Fig. 6). Moreover, we were able to construct *in vitro* model of CSCs, by which not only acquiring enough number of CSCs, but also the long term culture of the cells is possible.

Although the mechanisms and major factors remains to be elucidated, our results may provide new insights into the origin of the CSCs, and useful tools to the cancer researchers for development of CSC-targeted cancer therapies.

## Materials and Methods

### Cell culture and reagents

Cryopreserved B16F10 melanoma cells and NIH3T3 cells were purchased from ATCC (Rockville, MD, USA), and J1 mouse embryonic stem cells (J1 mES) were obtained from KRIBB (Daejeon, South Korea). B16F10 and NIH3T3 cells were grown in Dulbecco’s Modified Eagle’s Medium (DMEM; Hyclone, Logan, UT, USA) supplemented with 10% fetal bovine serum (FBS; Hyclone) and penicillin/streptomycin (100 U/ml; Hyclone) at 37 °C in a humidified incubator (5% CO_2_). J1 mES cells were grown in DMEM supplemented with 20% FBS, penicillin/streptomycin (100 U/ml), 1% non-essential amino acid (Hyclone), 1% L-glutamin (Hyclone), 0.1% β-mercaptoethanol (Gibco, Gaithersburg, MD, USA), and 5 × 10^5^ leukemia inhibitory factor (LIF; Millipore, Billerica, MA, USA) at 37 °C in a humidified incubator (5% CO_2_). J1 mES cells were cultured on a 0.1% gelatin-coated plate at 37 °C in a humidified atmosphere (5% CO_2_). Thiazolyl blue tetrazolium blue (MTT), cisplatin, mitomycin C, verapamil, and Hoechst 33342 were purchased from Sigma (St. Louis, MO, USA).

### Isolation of tumor-derived proteins from B16F10 melanoma cells

Mouse melanoma cells were harvested using DMEM containing a protease inhibitor and a phosphatase inhibitor. We performed sonication to obtain proteins of cancer cells and centrifuged. We collected the supernatants and filtered them using the syringe filter.

### Induction of colony formation

NIH3T3 cells were seeded in 24-well tissue culture plates at a density of 2 × 10^4^ cells/well. In the following day, the cells were treated with designated concentrations of extracted proteins from B16F10 melanoma cells. After 48 h incubation, each well of the plates was observed using a microscope.

### Cell viability assay

The cells were seeded in 96-well tissue culture plates at a density of 1 × 10^3^ cells/well. After the time was set to 24 or 48 h, 0.5 mg/ml of MTT was added to each well containing 100 μl of medium, and the cells were incubated at 37 °C for 4 h. The medium was removed, and 100 μl of Dimethyl Sulfoxide (DMSO) was added. After shaking for 5 min to mix thoroughly the formazan with the DMSO, absorbance was measured on a spectrum microplate spectrophotometer at 550 nm.

### Spheroid formation culture

The medium of the wells in which the colonies were formed was removed, and 100 μl of 1xPBS was added. The colony was isolated using a yellow tip. The isolated colonies were transferred to ultra-low attachment plates and cultured at 37 °C in a humidified incubator (5% CO_2_). In case of a subculture, the colonies were harvested, treated with trypsin (0.25%)–EDTA for 1 min, and added with a medium. After centrifugation at 1,200 rpm at 4 °C for 2 min, the colonies were re-suspended using the complete mES medium. The colonies in a single-cell condition were seeded on ultra-low attachment plates and cultured at 37 °C in a humidified incubator (5% CO_2_).

### Soft agar assay

A bottom, cell-free layer of 1% novel agar and a cell culture medium containing 20% FBS was plated in 12-well tissue culture plates and allowed to solidify at room temperature on a clean bench. A top layer of 0.7% novel agar and a culture medium containing 20% FBS, including 1 × 10^3^ colony cells, was plated over the solid bottom layer. The plates were incubated at 37 °C and 5% CO_2_ for 10–14 days until colonies grew to about 100 μm in diameter.

### Alkaline phosphatase staining assay

J1 mES cells were seeded in a 0.1% gelatin-coated 24-well tissue culture plate at a density of 1 × 10^3^ cells/well and cultured for five days. The cells were then fixed in 4% paraformaldehyde for 2 min and washed five times with 1xTBST. The washed cells were stained by an alkaline phosphatase (AP) staining solution (Millipore, Billerica, MA, USA) mixture (FRV:NAphthol:water = 2:1:1) for 30 min in the dark. Then, the cells were washed with 1xPBST and observed at 1xPBS.

### Cell cycle analysis

The cells were seeded in 6-well tissue culture plates at a density of 1 × 10^5^ cells/well. After one and two days, the cells were harvested by centrifugation at 1200 rpm for 3 min and washed three times in 1xPBS. The cells were resuspended in 200 μl of cold 70% EtOH and incubated at −20 °C for 3 h. After centrifugation at 1200 rpm for 3 min, the cells were washed in 1xPBS and harvested. The cells were treated with 50 μl of a 100 μg/ml stock of RNase and 200 μl of 50 μg/ml stock of propidium iodide. The cell cycle distribution was measured by the flow cytometry.

### Chemotherapy resistance assay

The cells were seeded in 96-well tissue culture plates at a density of 1 × 10^3^ cells/well. On the following day, the cells were treated with designated concentrations of cisplatin and mitomycin C. After 24 h incubation, 0.5 mg/mL of MTT was added to each well containing 100 μl of medium, and the cells were incubated at 37 °C for 4 h. The medium was removed, and 100 μl of DMSO was added. After shaking for 5 min to mix thoroughly the formazan with DMSO, absorbance was measured on the spectrum microplate spectrophotometer at 550 nm.

### Reverse transcription-polymerase chain reaction

RNA was isolated from cells using RNeasy mini kit (Qiagen, Valencia, CA, USA) and cDNAs were synthesized using a PrimeScript^TM^ RT reagent kit (Takara, Shiga, Japan) according to the manufacturer’s instruction. Reverse transcription–polymerase chain reaction (RT-PCR) was performed with the EmeraldAmp PCR Master Mix using the Thermal Cycler Dice PCR machine (Takara, Shiga, Japan). The primers used were as follows: Oct4 (forward 5′-AGCTGCTGAAGCAGAAGAGG-3′, reverse 5′-TGGGAAAGGTGTCCCTGTAG), Sox2 (forward 5′-AGAACCCCAAGATGCACAAC-3′, reverse 5′-ATGTAGGTCTGCGAGCTGGT-3′), c-Myc (forward 5′-GCCCAGTGAGGATATCTGGA-3′, reverse 5′-ACTGAGGGGTCAATGCACTC-3′), Klf4 (forward 5′-CAGCTTCATCCTCGTCTTCC -3′, reverse 5′-CGCCTCTTGCTTAATCTTGG -3′), CD44 (forward 5′-GAAAGGCATCTTATGGATGTGC -3′, reverse 5′-CTGTAGTGAAACACAACACC -3′), CD133 (forward 5′-CTCATGCTTGAGAGATCAGGC -3′, reverse 5′-CGTTGAGGAAGATGTGCACC -3′), ABCG2 (forward 5′-CAGCAGCTCTTCGACTTCCA -3′, reverse 5′-ATCCGCAGGGTTGTTGTA -3′), and β-actin (forward 5′-CCTAAGGCCAACCGTGAA -3′, reverse 5′-CCGCTCGTTGCCAATAGT -3′).

### Flow cytometry analysis

IgG2 isotype-FITC (Invitrogen, Waltham, MA, USA), CD44-FITC (Invitrogen), IgG1 isotype-PE (Invitrogen), and CD133-PE (Invitrogen) were used to measure the CD44 and CD133 positive population. To analyze the relative CD44 and CD133 expression level in colonies, 2 × 10^5^ cells were incubated with 100 μl of flow cytometry buffer containing 2 μl IgG2 isotype-FITC, CD44-FITC and 5 μl IgG1 isotype-PE, CD133-PE for 30 min at 4 °C in the dark. The cells were washed two times with 1xPBS and resuspended with 100 μl of flow cytometry buffer to measure the flow cytometry.

### Drug efflux assay

Colonies were seeded on ultra-low attachment plates at 1 × 10^3^ cells/well and cultured at 37 °C in a humidified incubator (5% CO_2_). After three days, the colonies were treated with 200 μM verapamil and incubated at 37 °C for 20 min. Then, Hoechst 33342 dye was added to a final concentration of 2 μg/ml in the presence or absence of verapamil. The colonies were incubated at 37 °C for 30 min in the dark and observed using a fluorescent microscope.

### Xenograft assay

Male BALB/cAnNTac nude mice were obtained from Raon Bio. All mice used in the study were six to eight weeks of age. The mice were injected subcutaneously (s.c.) with 1 × 10^6^ NIH3T3 cells and induced colonies in Hank’s balanced salt solution (100 μl). The mice were weighed, and the tumors were monitored two to three times per week in two diameters using calipers 10 days after injection. The tumor volumes were determined using A^2^ × B × 0.5, where A is the shortest diameter and B is the longest diameter. All animal experiments were approved by the Institutional Animal Care and Use Committee of the Korea Research Institute of Bioscience and Biotechnology and were followed the guidelines of the Animal Care Committee of the Korea Research Institute of Bioscience and Biotechnology.

### NIH3T3-GFP stable cell line

To produce an NIH3T3-Green Fluorescent Protein (GFP) stable cell line, the pEGFP-C2 plasmid vector was cut with the *Eco*RI restriction enzyme to a linear form. The linear pEGFP-C2 plasmid was transfected into NIH3T3 cells on 24-well tissue culture plates using the Lipofectamine^TM^ 3000 reagent according to the manufacturer’s instruction, and the cells were incubated for 48 h. The medium was removed and replaced with a fresh complete DMEM medium, with G418 as the selective marker. The transfected cells were transferred to 96-well tissue culture plates while maintaining the G418 medium condition, and this step was repeated to isolate the NIH3T3-GFP positive cells. The NIH3T3-GFP positive cells were observed with a fluorescent microscope.

### Co-culture NIH3T3-GFP cells and B16F10 melanoma cells

NIH3T3-GFP cells were co-cultured with B16F10 melanoma cells at a ratio of 9:1, 5:5 and 1:9 for 48 h in 37 °C, 5% CO_2_ humidified incubator. The number of total cells is 2 × 10^5^ on each well of 6 well pates. Mixed-cells were observed at 24 h and 48 h using a fluorescence microscope.

### Culture of NIH3T3 cells using B16F10 melanoma cells-conditioned medium

B16F10 melanoma cells were cultured with Dulbecco’s Modified Eagle’s Medium (DMEM; Hyclone, Logan, UT, USA) supplemented with 10% fetal bovine serum (FBS; Hyclone) and penicillin/streptomycin (100 U/ml; Hyclone) at 37 °C in a humidified incubator (5% CO_2_) for 48 h in 37 °C, 5% CO_2_ humidified incubator. Next, supernatant was collected in tube and it was filtered using the syringe filter. The supernatant of melanoma cells was mixed with fresh compete medium at a ratio of 1:9, 5:5, and 10:0. Then, NIH3T3 cells were cultured with each mixed medium for 48 h in 37 °C, 5% CO_2_ humidified incubator.

### Statistical analysis

All experiments were repeated at least three times. Data are presented as means ± standard deviation. Analyses were performed with Student’s t test, and the level of significance was set to the probability of <0.05 (*P < 0.05; **P < 0.01; ***P < 0.001).

## Supplementary information

Supplementary figures
