# Supplementary material for: Extracellular Microenvironmental Change by B16F10 Melanoma-derived Proteins Induces Cancer Stem-like Cell Properties from NIH3T3 Cells
Source: Sci Rep. 2019 Nov 14;9:16757. doi: 10.1038/s41598-019-53326-8 (PMC6856526; doi:10.1038/s41598-019-53326-8)
Supplement: Supplementary file 1 — Supplementary figures [file 41598_2019_53326_MOESM1_ESM.pdf]

Supplementary Figures

Extracellular Microenvironmental Change by B16F10 Melanoma-derived Proteins Induces Cancer Stem-like Cell Properties from NIH3T3 Cells

Soon Yong Park, Dong Gwang Lee, Ara Jo, Hyeongrok Choi, Joo Eon Lee,

Ae Jin Jeong, Sun-Hee Leem, Woojin Jun, Sangin Shim, Sang-Kyu Ye,

Jeong-Ki Min, Jin Woong Chung

Supplementary Figure S1.

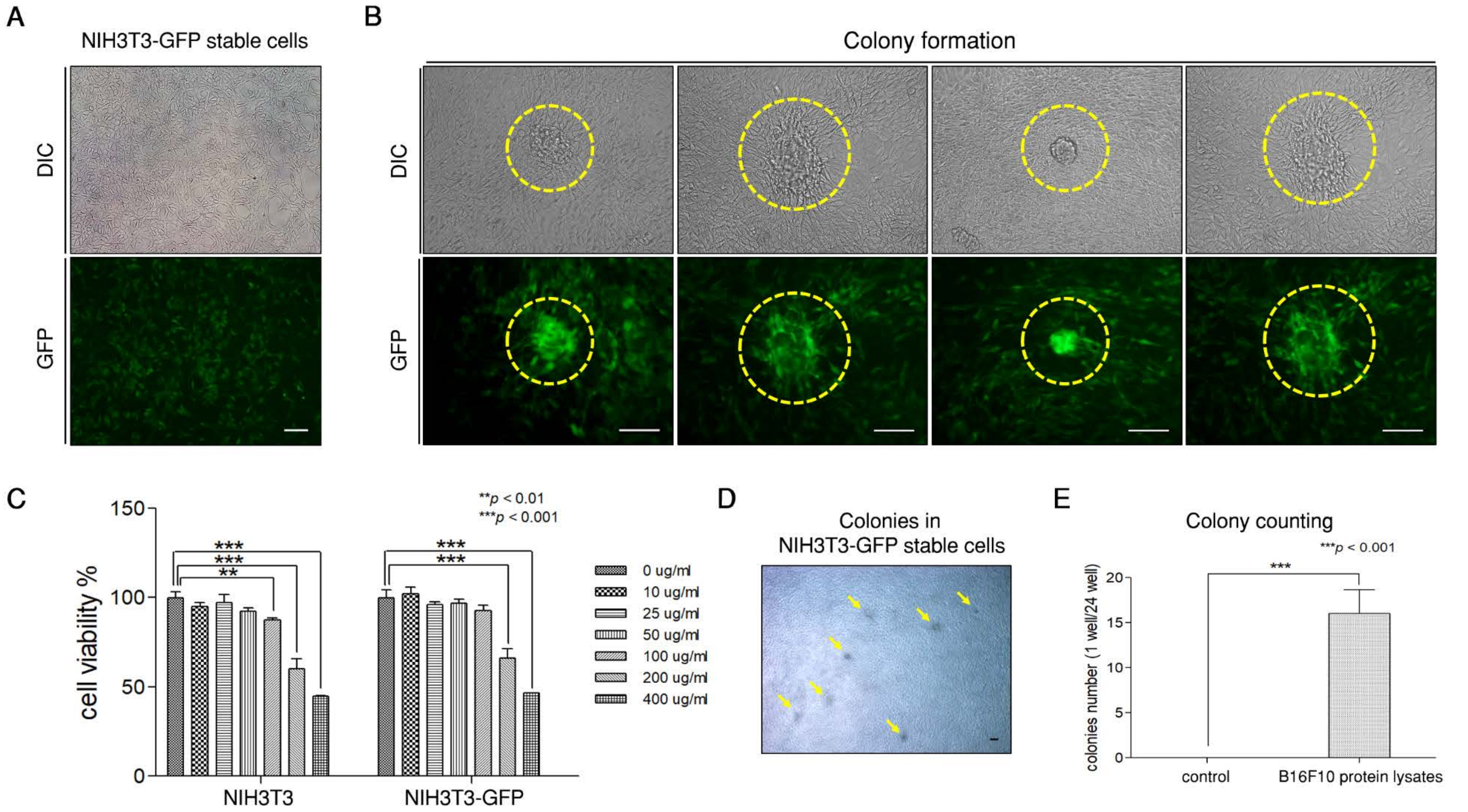

Supplementary Fig. S1. B16F10-derived proteins can induce GFP-positive colony formation from NIH3T3-GFP stable cells. (A) The NIH3T3 cells were transfected with the linear form of the pEGFP-C2 plasmid using the Lipofectamine^{TM} 3000 reagent. Then, the NIH3T3-GFP stable cells were isolated using complete DMEM, which contains G418. (B) The NIH3T3-GFP stable cells were treated with 50 mg/ml of B16F10-derived proteins for 48 h. The GFP-positive colonies were observed with a fluorescent microscope. (C) The NIH3T3 and NIH3T3-GFP stable cells were treated with a designated concentration of B16F10-derived proteins (0, 10, 25, 50, 100, 200, and 400 mg/ml). After 48 h, the MTT assay was performed, as described in the Materials and Methods section. Cell viability did not have a significant difference until 50 mg/ml was reached. However, cell viability decreased in a concentration-dependent manner from 100 mg/ml and over. (D, E) About 12–18 colonies were induced in one well of 24-well plates from the NIH3T3-GFP stable cells, similar to the generation ratio of wild-type NIH3T3 cells (yellow arrow). These results are the averages of three independent experiments. Scale bar: 100 μm.

Supplementary Figure S2.

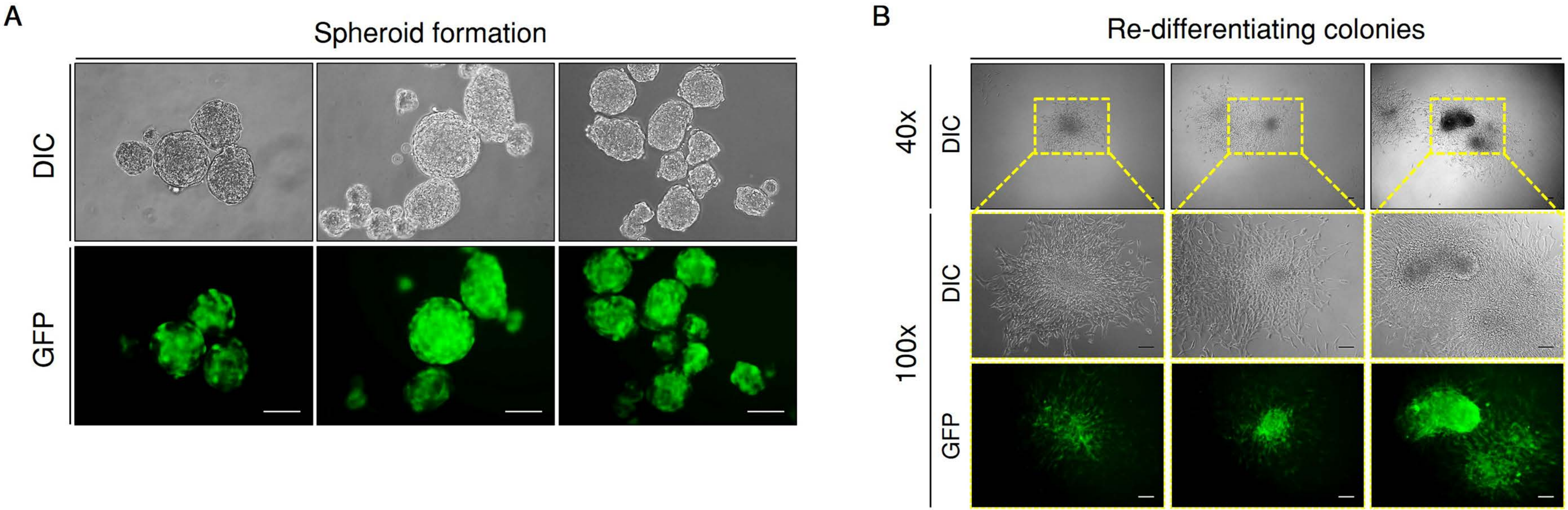

Supplementary Fig. S2. GFP-positive induced colonies acquire the properties of stem cells. (A) The GFP-positive induced colonies could form a spheroid morphology and grow on ultra-low attachment plates in the mES medium. (B) Anchorage independently cultured GFP-positive induced colonies were transferred to normal culture plates, and the colonies were differentiated into normal cells in a time-dependent manner. These results are the averages of three independent experiments. Scale bar: 100 μm.

Supplementary Figure S3.

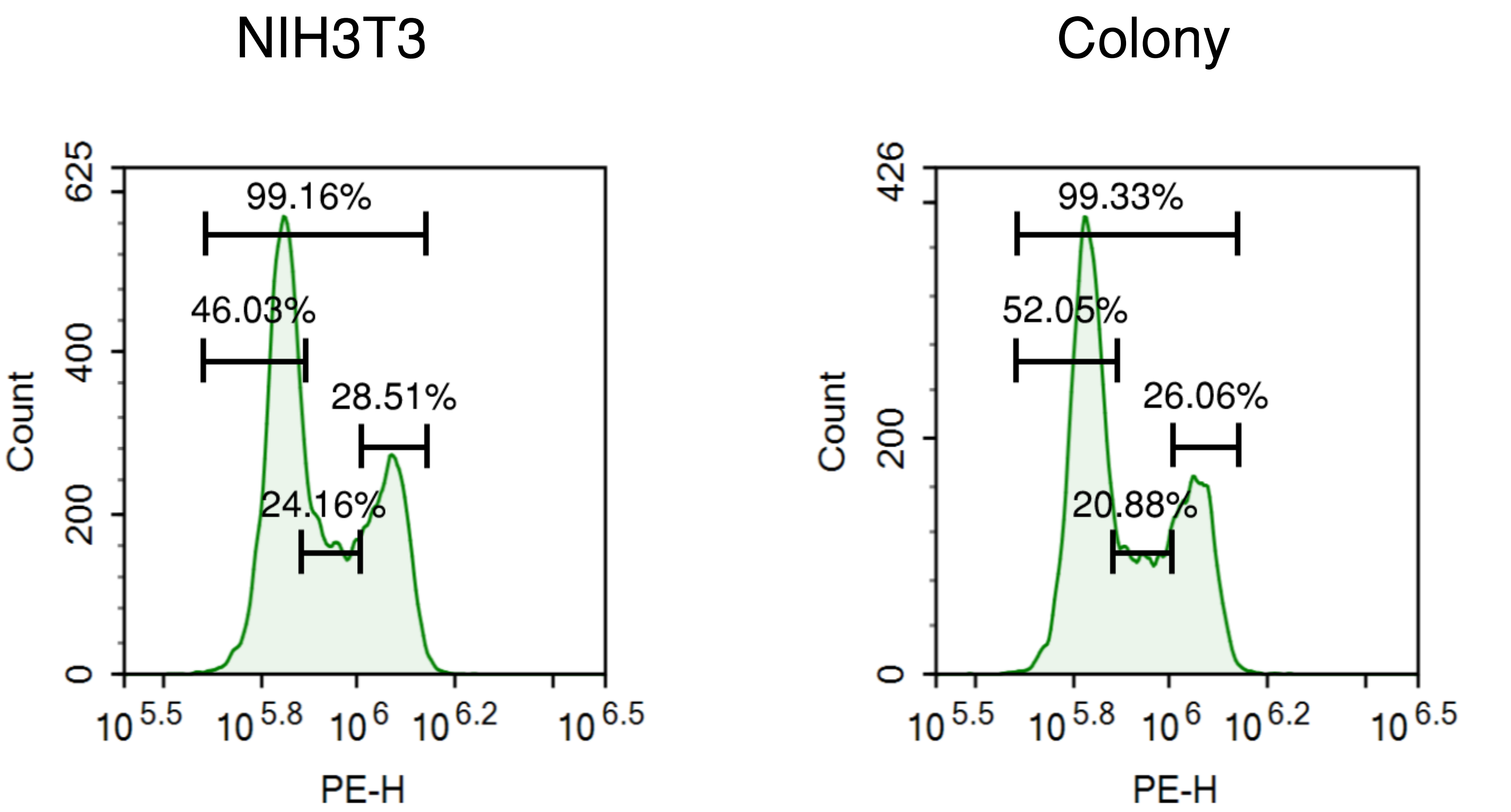

Supplementary Fig. S3. Histograms of cell cycle distribution analysis of NIH3T3 and induced-colonies . Colonies maintained a higher proportion of the G0/G1 phase and a lower proportion of the S phase and G2/M than the NIH3T3 cells.

Supplementary Figure S4.

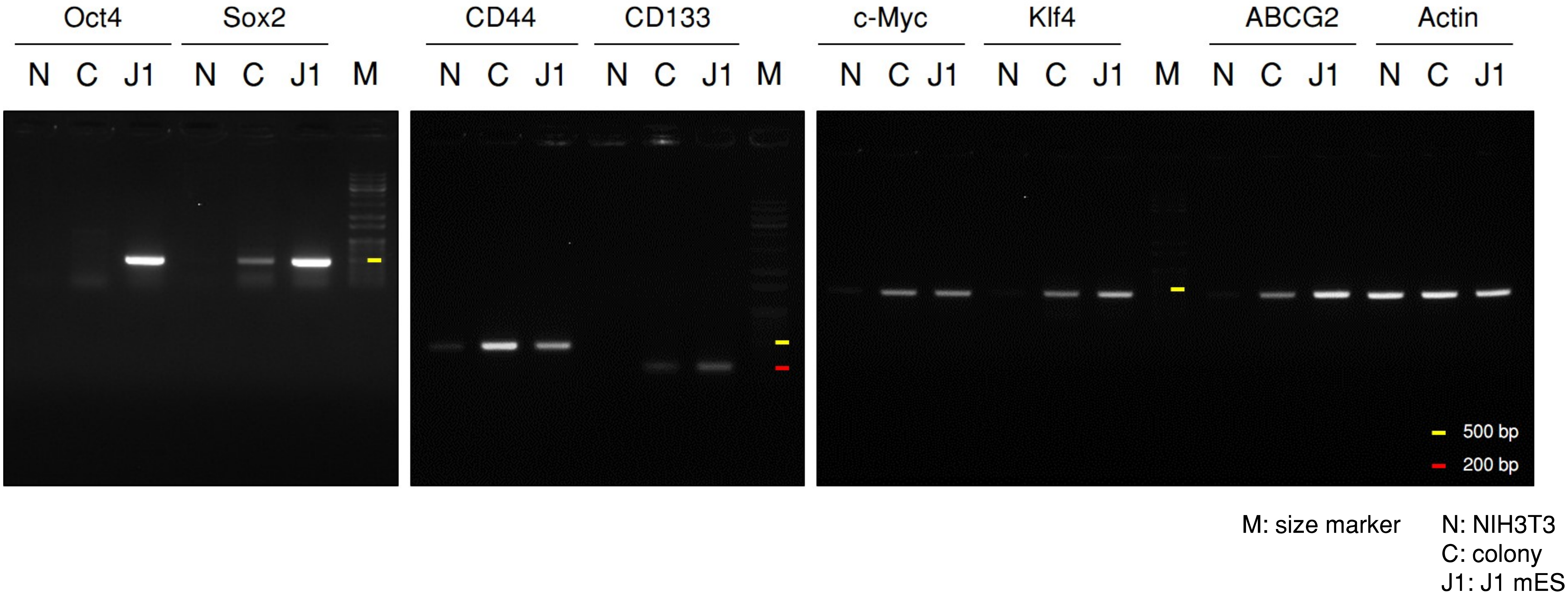

Supplementary Fig. S4. Induced colonies express various CSC markers. Induced colonies expressed CSC markers such as Oct4, Sox2, c-Myc, Klf4, CD44, CD133 and ABCG2. (Product size - Oct4: 466 bp, Sox2: 465 bp, c-Myc: 449 bp, Klf4: 449 bp, CD44: 450 bp, CD133: 222 bp, ABCG2: 449 bp, Actin: 429 bp)

Supplementary Figure S5.

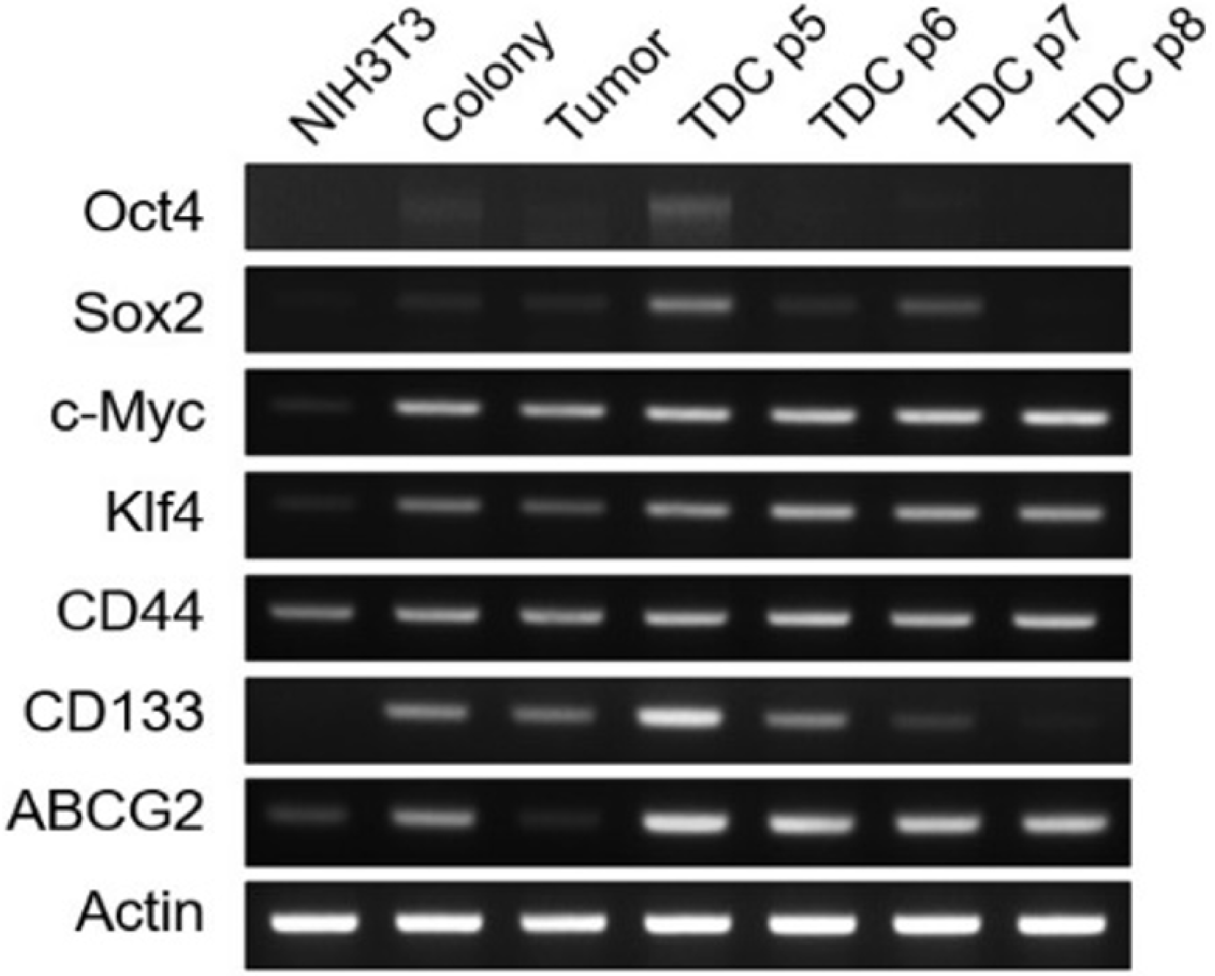

Supplementary Fig. S5. Oct4, Sox2, and CD133 as CSC marker were significantly decreased as the passage of cells increased. The expressions of stem cell markers such as Oct4, CD133, and Sox2 decreased rapidly while the expression levels of oncogenes including c-Myc, Klf4 and ABCG2 were maintained, as the passage of the tumor-derived cells (TDCs) increased. This observation indicates that the induced colonies from the NIH3T3 cells maintains the tumorigenic properties such as solid tumor initiation, growth, and progression *in vivo*, still retaining their differentiating capacities (full-length gels are presented in Supplementary Fig. S6).

Supplementary Figure S6.

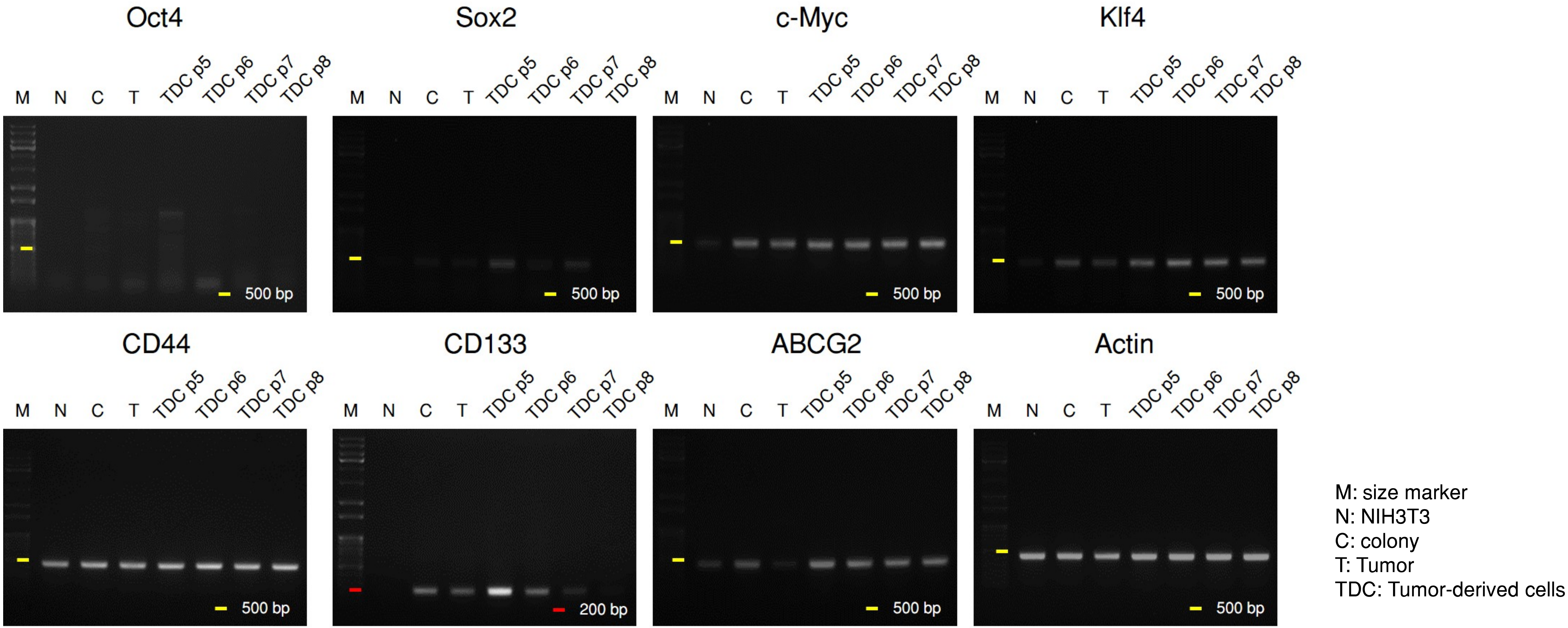

Supplementary Fig. S6. Induced colonies, formed tumor *in vivo* from induced colonies and tumor-derived cells show different expression level of CSC markers. NIH3T3 cells, induced colonies, tumor and tumor-derived cells show the different expression level of various CSC markers. (Product size - Oct4: 466 bp, Sox2: 465 bp, c-Myc: 449 bp, Klf4: 449 bp, CD44: 450 bp, CD133: 222 bp, ABCG2: 449 bp, Actin: 429 bp)

Supplementary Figure S7.

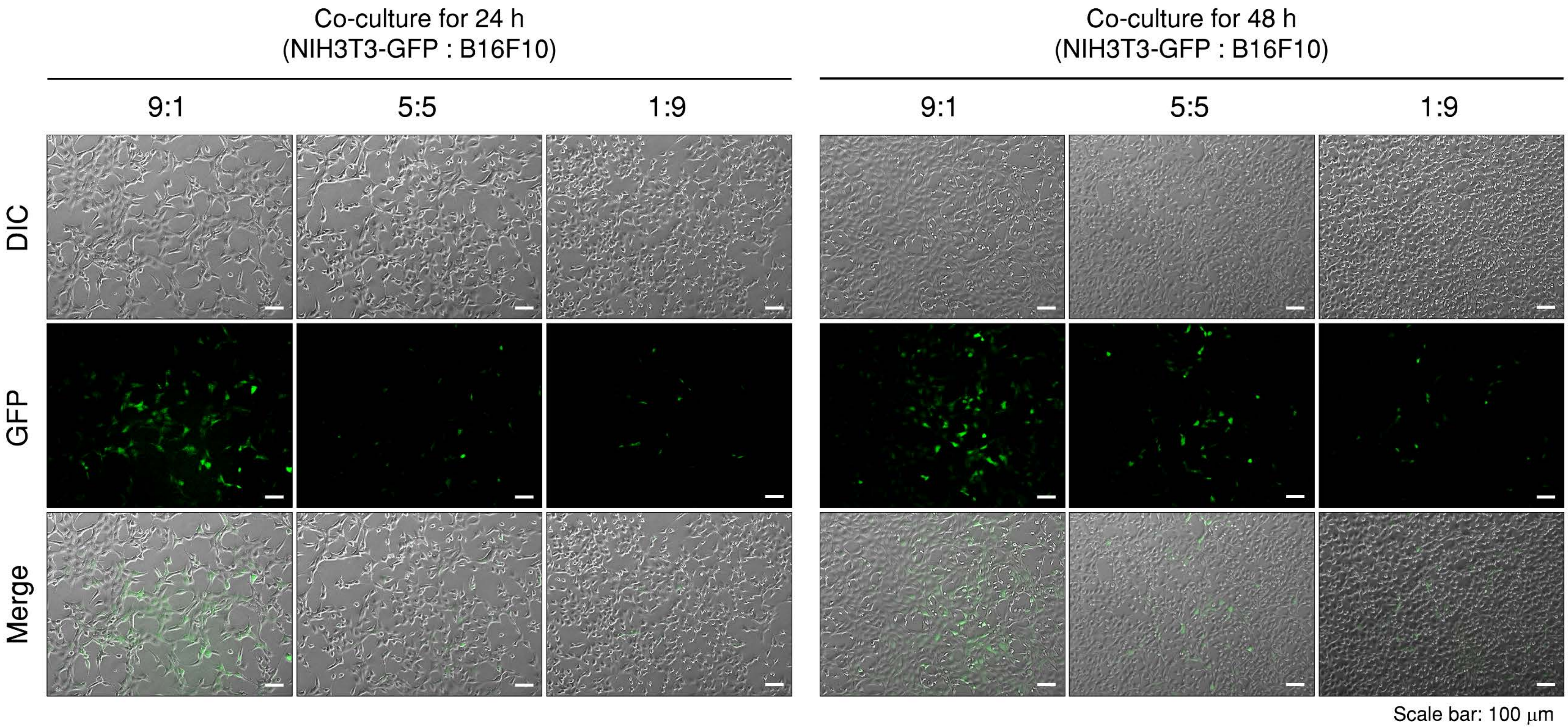

Supplementary Fig. S7. NIH3T3-GFP cells co-cultured with B16F10 melanoma cells can not induce colony formation . NIH3T3-GFP cells were co-cultured with B16F10 melanoma cells for 48 h in 37°C, 5% CO_2 humidified incubator. (ratio of NIH3T3-GFP cells and B16F10 melanoma cells = 9:1, 5:5 and 1:9, number of total cells: $2x10^5$ in 6 well pates). However, co-culturing with B16F10 could not transform NIH3T3-GFP cells into colony. These results are the averages of three independent experiments. Scale bar: 100 mm.

Supplementary Figure S8.

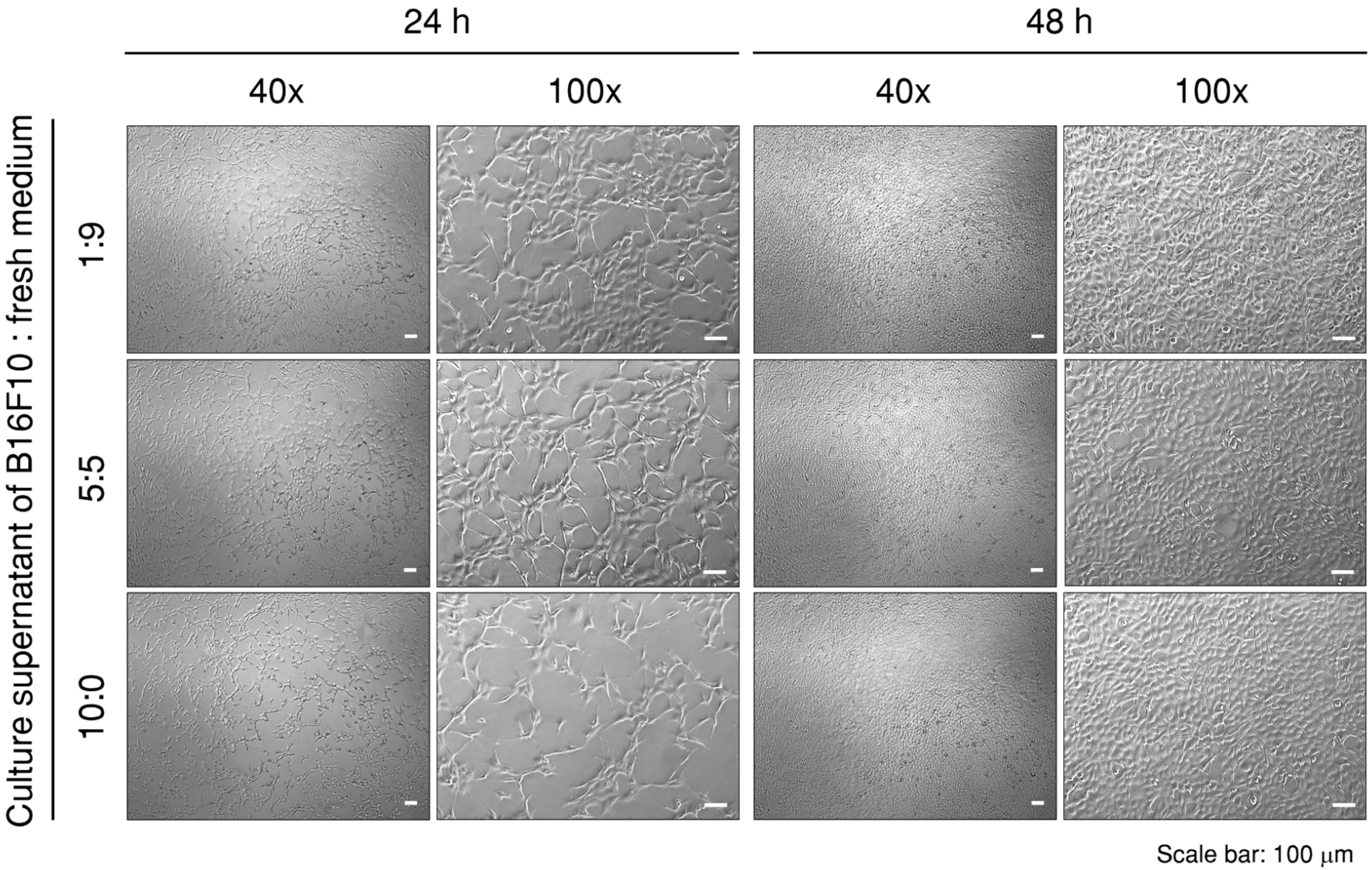

Supplementary Fig. S8. Culture supernatant of B16F10 melanoma cells can not induce colony formation . NIH3T3 cells were cultured with medium containing culture supernatant of B16F10 melanoma cells incubated for 48 h. NIH3T3 cells were incubated with three types of medium (ratio of culture supernatant of B16F10 cells and fresh complete medium = 1:9, 5:5 and 10:0) for 48 h in 37°C, 5% CO_2 humidified incubator. However, culture supernatant of B16F10 could not transform NIH3T3 cells into colony. These results are the averages of three independent experiments. Scale bar: 100 mm.
